# Connexin 43 Modulation in Human Chondrocytes, Osteoblasts and Cartilage Explants: Implications for Inflammatory Joint Disorders

**DOI:** 10.3390/ijms25158547

**Published:** 2024-08-05

**Authors:** Elena Della Morte, Chiara Giannasi, Alice Valenza, Francesca Cadelano, Alessandro Aldegheri, Luigi Zagra, Stefania Niada, Anna Teresa Brini

**Affiliations:** 1 Laboratory of Biotechnological Applications, IRCCS Istituto Ortopedico Galeazzi, 20157 Milan, Italy; elena.dellamorte@grupposandonato.it (E.D.M.); chiara.giannasi@unimi.it (C.G.); alice.valenza@virgilio.it (A.V.); francesca.cadelano@unimi.it (F.C.); anna.brini@unimi.it (A.T.B.); 2 Department of Biomedical, Surgical and Dental Sciences, University of Milan, 20129 Milan, Italy; 3 Hip Department, IRCCS Istituto Ortopedico Galeazzi, 20157 Milan, Italy; aldegherialess@gmail.com (A.A.); luigi.zagra@fastwebnet.it (L.Z.)

**Keywords:** Connexin 43, chondrocytes, osteoblasts, cartilage explants, TNFα, IL-1β, synovial fluid, osteoarthritis

## Abstract

Connexin 43 (Cx43) is crucial for the development and homeostasis of the musculoskeletal system, where it plays multifaceted roles, including intercellular communication, transcriptional regulation and influencing osteogenesis and chondrogenesis. Here, we investigated Cx43 modulation mediated by inflammatory stimuli involved in osteoarthritis, i.e., 10 ng/mL Tumor Necrosis Factor alpha (TNFα) and/or 1 ng/mL Interleukin-1 beta (IL-1β), in primary chondrocytes (CH) and osteoblasts (OB). Additionally, we explored the impact of synovial fluids from osteoarthritis patients in CH and cartilage explants, providing a more physio-pathological context. The effect of TNFα on Cx43 expression in cartilage explants was also assessed. TNFα downregulated Cx43 levels both in CH and OB (−73% and −32%, respectively), while IL-1β showed inconclusive effects. The reduction in Cx43 levels was associated with a significant downregulation of the coding gene GJA1 expression in OB only (−65%). The engagement of proteasome in TNFα-induced effects, already known in CH, was also observed in OB. TNFα treatment significantly decreased Cx43 expression also in cartilage explants. Of note, Cx43 expression was halved by synovial fluid in both CH and cartilage explants. This study unveils the regulation of Cx43 in diverse musculoskeletal cell types under various stimuli and in different contexts, providing insights into its modulation in inflammatory joint disorders.

## 1. Introduction

Connexins (Cx) constitute a family of integral membrane proteins forming hemichannels that facilitate cellular communication with the extracellular environment. The aggregation of two hemichannels between adjacent cells results in gap junctions (GJ), allowing the exchange of ions and small molecules up to 1.2 kDa. These interactions play a pivotal role in regulating cell survival, growth and metabolism [1]. Each Cx consists of nine main domains, among which the N-terminus, two extracellular loops and four transmembrane helical domains are highly conserved between different isoforms. In contrast, the cytoplasmic loop domain and the C-terminus domain show variability in length and sequence [2]. Cx are ubiquitously expressed in different cell types among which are neurons, cardiomyocytes, adipocytes, osteocytes, osteoblasts and chondrocytes. In particular, the human genome contains about 21 genes that encode related Cx isoforms, generally classified according to their molecular weight (from 26 to 60 kDa) [3].

Notably, Connexin 43 (Cx43), a well-studied Cx type, remains abundant in various human tissues, particularly in the musculoskeletal system [4]. Expressed throughout the musculoskeletal system, Cx43 plays a crucial role in its development, maintenance of homeostasis and repair processes [5]. In fact, osteocytes, osteoblasts and osteoclasts, mainly through GJ, communicate and arrange themselves assembling a large cell network. Both hemichannel and GJ contribute to the functionality of the skeleton; hemichannels guide osteocyte survival, endocortical bone resorption and periosteal bone apposition and GJ mediate remodeling. Beyond its role in intercellular communication, Cx43 acts as a transcriptional factor, influencing crucial cellular processes such as cell cycle progression [6] and the n-cadherin pathway [7]. Noteworthy findings show its involvement in osteogenesis, where a reduction in the *GJA1* gene (encoding Cx43) leads to delayed bone development [8]. Patients with mutations in *GJA1* present craniofacial abnormalities (such as skull hyperostosis, a pointed nose and enamel hypoplasia) [9]. Cx43 is additionally expressed by chondrocytes and recent studies have shown that its deficiency, particularly in the carboxy terminal (CTD) domain, alters the structure of cartilage and the typical phenotype of chondrocytes. Furthermore, CTD-deficient chondrocytes have a decreased ability to communicate through GJ channels, and display defective cellular proliferation and decreased levels in the synthesis of the components of the extracellular matrix [10]. 

In this context, our research focuses on understanding the modulation of Cx43 in response to external stimuli in different musculoskeletal cell types. Our previous work demonstrated that Tumor Necrosis Factor alpha (TNFα) induces a proteasome-mediated degradation of Cx43 in articular chondrocytes (CH) [11]. 

Our current study expands on the exploration of the impact of TNFα and Interleukin 1 beta (IL-1β) stimulation on Cx43 expression in primary chondrocytes and paired osteoblasts. Additionally, providing a more physio-pathological stimulus, we examine the impact of synovial fluids collected from severe osteoarthritis patients on Cx43 modification in CH. Finally, we used cartilage explants to investigate Cx43 modulation in CH in the presence of the proper extracellular matrix. By elucidating the modulation of Cx43 in diverse musculoskeletal cell types under various stimuli, our research aims to contribute to a deeper understanding of the role of Cx43 in inflammatory and/or degenerative joint disorders, such as osteoarthritis. 

## 2. Results

**Figure 1:**
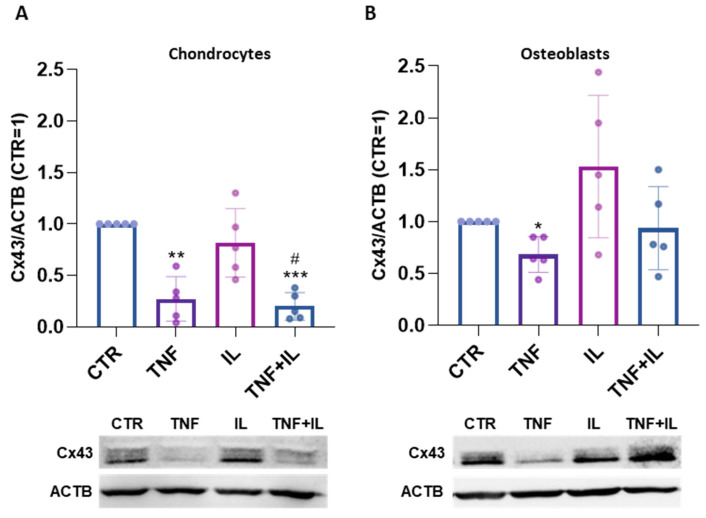
(**A**,**B**). Expression of Cx43 in TNFα and/or IL-1β-stimulated human articular chondrocytes (CHs) (**A**) and human osteoblasts (OBs) (**B**) at day 3, analyzed using Western blot. Specific bands were quantified through Image Lab Software v 6.1 (Bio-Rad, Milan, Italy) and data (*n* = 5 independent experiments/donors, each indicated by distinct dots) were normalized on ACTB and expressed as relative values (CTR = 1). The panels below show representative immunoblots. Statistical analysis was performed via one-way analysis of variance (ANOVA) using Tukey’s post hoc test. Data are shown as mean ± SD. Significances vs. CTR are shown as * *p* ≤ 0.05, ** *p* < 0.01 and *** *p* < 0.001; vs. IL # *p* ≤ 0.05.

Chondrocytes (CH) and osteoblasts (OB) underwent a three-day treatment with TNFα and/or IL-1β to investigate the impact of these two distinct cytokines on Cx43 protein expression. Following the TNFα stimulus, both CH and OB exhibited a reduction in the expression of Cx43 (−73% and −32%, respectively). Similarly, the co-administration of TNFα and IL-1β in CH resulted in a comparable decrease in Cx43 expression. Notably, IL-1β alone did not exhibit discernible effects on both cell types. Moreover, the simultaneous administration of IL-1β and TNFα did not result in any distinct outcomes in OB [Figure 1A,B]. 

**Figure 2:**
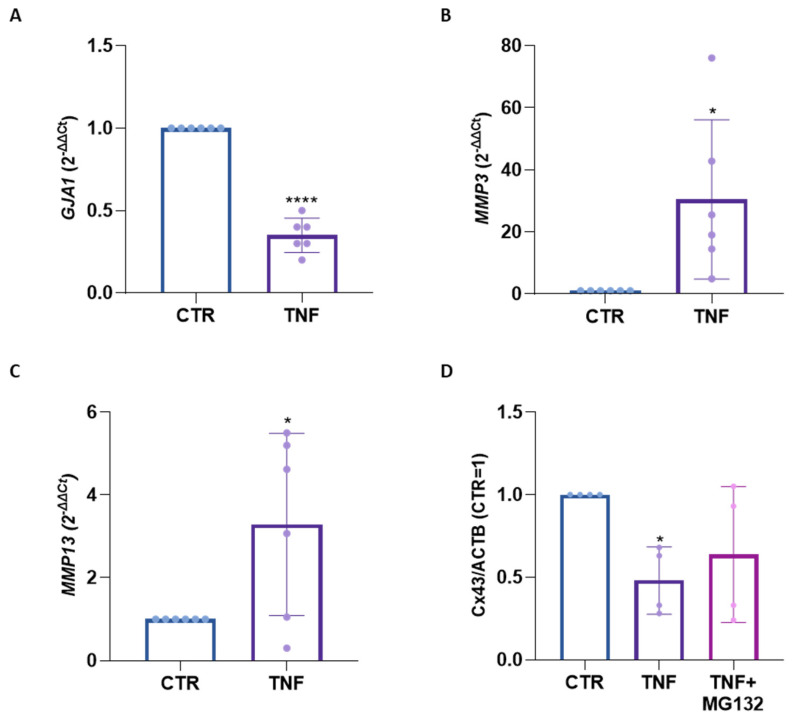
(**A**–**C**). Gene expression of GJA1 (**A**), MMP3 (**B**) and MMP13 (**C**) in TNFα-stimulated OB at day 1 analyzed using real-time PCR. Data (*n* = 6 independent experiments/donors, each indicated by distinct dots) are expressed as 2^−ΔΔCt^ (TBP was used as a housekeeping gene). (**D**). Expression of Cx43 in OB pre-treated for 1 h with MG-132 and then stimulated with TNFα for 3 days, analyzed using Western blot. Specific bands were quantified through Image Lab Software v 6.1 (Bio-Rad, Milan, Italy) and data (*n* = 4 independent experiments/donors) were normalized on ACTB and expressed as relative values (CTR = 1). Statistical analyses were performed using paired *t*-test (**A**–**C**) or one-way analysis of variance (ANOVA) using Tukey’s post hoc test (**D**). Data are shown as mean ± SD. Significance vs. CTR is shown as * *p* ≤ 0.05 and **** *p* < 0.0001.

In our earlier research on TNFα stimulated articular chondrocytes, the reduction in Cx43 levels did not occur at the transcriptional level (as also confirmed in the current study; Supplementary Figure S1); rather, it was attributed to proteasomal degradation [11]. Differently, in this study, we identified that both mechanisms are involved in the reduction in Cx43 in osteoblasts. In OB treated with TNFα for one day, while *MMP* expression was significantly enhanced [Figure 2B,C], the expression of *GJA1* gene, which encodes Cx43, was downmodulated [Figure 2A]. In detail, TNFα treatment led to a 65% reduction in *GJA1* gene expression in OB [Figure 2A]. To explore the hypothetical role of the proteasome, OB were pre-treated for one hour with the proteasome inhibitor MG132 (the efficacy of which in inhibiting the proteasome at the selected concentration is shown in Supplementary Figure S2) before exposure to TNFα. This led to a partial restoration of Cx43 expression [Figure 2D]. 

**Figure 3:**
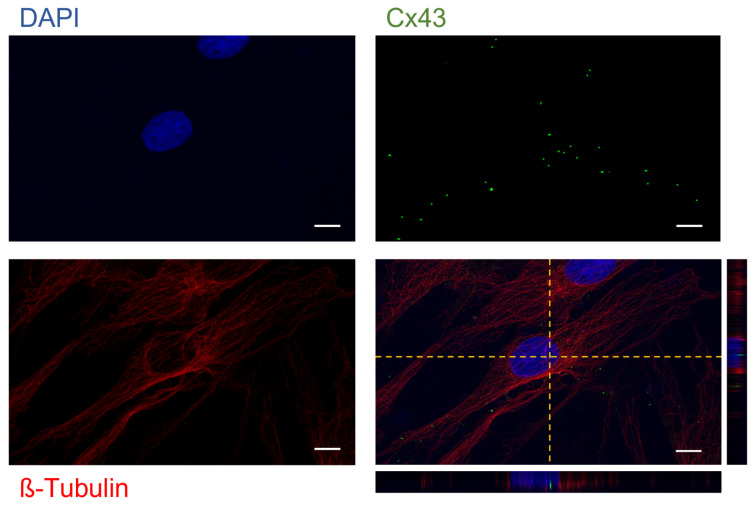
Laser scanning confocal microscopy of OB; Cx43 and β-Tubulin were revealed with an Alexa Fluor^®^ 488 (Cx43) and 568 (β-tubulin) conjugated antibody (green and red respectively), while nuclei were stained with DAPI (blue) (magnification 63×). The scale bar indicates 10 µm and the orthogonal views (yellow dashed lines) were obtained using Fiji software (ImageJ 1.51).

At last, we looked for the presence of Cx43 both at the membrane and nucleus levels, as previously performed in CH [11]. The classical punctiform Cx43 signal was observed throughout the cell. Cx43 was also detectable in the perinuclear region and at the nucleus level, as revealed through the co-localization of Cx43 green signals with DAPI staining [Figure 3; Supplementary Figure S3].

**Figure 4:**
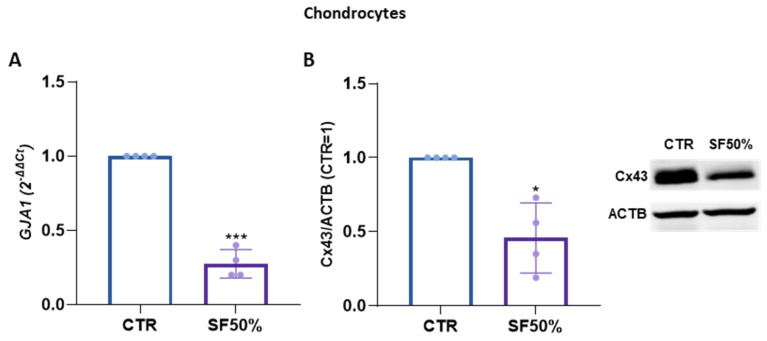
(**A**). Gene expression of GJA1 in CH treated with synovial fluid (SF50%) for 1 day analyzed using real-time PCR. Data (*n* = 4 independent experiments/donors, each indicated by distinct dots) are expressed as 2^−ΔΔCt^ (TBP was used as a housekeeping gene). (**B**). Expression of Cx43 in CH treated with SF50% for 3 days analyzed using Western blot. A representative immunoblot is shown. Specific bands were quantified through Image Lab Software v 6.1 (Bio-Rad, Milan, Italy) and data (*n* = 4 independent experiments) were normalized on ACTB and expressed as relative values (CTR = 1). Data are shown as mean ± SD. Statistical analysis was performed using paired *t*-test. Significance vs. CTR is shown as * *p* ≤ 0.05 and *** *p* < 0.001.

Our results on Cx43 expression highlighted a different response to the two inflammatory cytokines highly present in the context of osteoarthritis. To mimic pathophysiological condition, synovial fluids from OA patients were added to CH that were maintained in culture for either one or three days. Interestingly, both the gene and protein expressions of Cx43 were significantly downregulated by synovial fluid. Specifically, Cx43 gene expression decreased by 73% [Figure 4A], while at the protein level the reduction was by 54% [Figure 4B]. 

**Figure 5:**
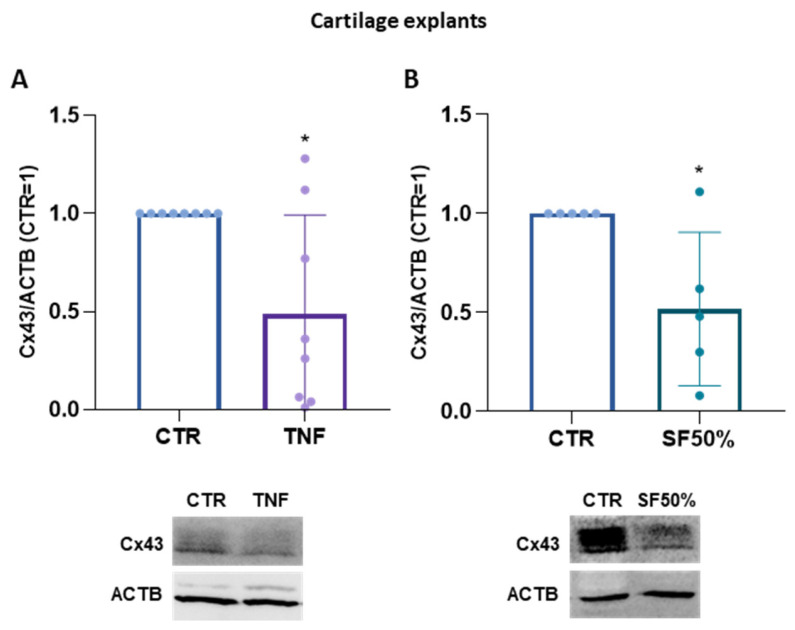
(**A**,**B**). Expression of Cx43 in cartilage explants treated with TNFα (**A**) or SF50% (**B**) for 3 days analyzed using Western blot. Representative immunoblots are shown. Specific bands were quantified through Image Lab Software v 6.1 (Bio-Rad, Milan, Italy) and data (*n* = 8 and *n* = 5 independent experiments/donors, each indicated by distinct dots) were normalized on ACTB and expressed as relative values (CTR = 1). Data are shown as mean ± SD. Statistical analysis was performed using paired *t*-test. Significance vs. CTR is shown as * *p* ≤ 0.05.

Up to now, our analyses of Cx43 have been conducted in 2D cultures, where both cell density and the extracellular environment significantly deviate from natural conditions; we therefore also decided to investigate the modulation of Cx43 in cartilage explants. Remarkably, we observed a significant reduction in Cx43 levels after 3 days of treatment with both TNFα (−51%) [Figure 5A] and synovial fluids (−48%) [Figure 5B], mirroring the effect observed in monolayer cultures.

## 3. Discussion

Connexins play a pivotal role in the development, homeostasis and plasticity of the skeletal system. Among the various connexins, Cx43 stands out as the most expressed one in gap junctions across bone cells and cartilage, making it a key focus of investigation [3]. Our previous work demonstrated that TNFα, a mediator implicated in articular diseases such as osteoarthritis (OA), downregulates Cx43 levels in chondrocytes through proteasome activation, impacting both gap junction numbers and nuclear Cx43 expression [11]. Given that degenerative processes in musculoskeletal diseases extend beyond cartilage, impacting all joint components [12], our study aims to investigate Cx43 modulation following treatment with two inflammatory cytokines, in the most relevant cell types of the osteochondral unit, namely chondrocytes and osteoblasts. Chondrocytes are the only cell type in articular cartilage, the tissue mostly involved in OA. In addition, osteoblasts represent a crucial cell type in the pathophysiology of the osteochondral unit, as supported by a growing body of evidence in the literature. During the onset and progression of osteoarthritis, these cells exhibit altered functionality and undergo changes affecting their metabolism and gene expression [13]. Moreover, CH and cartilage explants were also treated with synovial fluid in order to approach a more patho-physiological condition.

Firstly, we compare Cx43 modulation in chondrocytes (CH) and osteoblasts (OB) derived from the same donors after TNFα and IL-1β treatment. Both cytokines, crucial in OA development, are known to reduce the synthesis of proteoglycan components and type II collagen in chondrocytes. In addition, they lead to extracellular matrix (ECM) degradation by inducing collagenases and aggrecanases including MMP-1, MMP-3, MMP-13 and ADAMTS-4 and the production of proinflammatory mediators such as iNOS, COX-2 and PGE-2 synthase [14]. 

The influence of TNFα on Cx43 protein expression exhibited remarkable similarities between CH and OB. The downregulation of Cx43 by TNFα has been previously documented in CH [11], as well as in various other cell types including keratinocytes [15] and human corneal fibroblasts [16,17]. Of note, this effect has never been described in OB. Surprisingly, IL-1β did not induce any clear modulation of Cx43 in CH and OB. Though the heterogeneous effect of the two inflammatory cytokines is peculiar, it is known that TNFα and IL-1β exert different effects in different contexts. In synoviocytes, TNFα, in combination with IL-17, can increase the expression of the zinc and metallothionine transporter, while IL-1β does not lead to the same effect, thus determining a lower cadmium uptake in synoviocytes treated with IL-1β/IL-17 [18]. In committed myoblasts, the two cytokines exert opposite effects on myogenin expression and localization at the nuclear level [19]. In addition, the proteomic analyses highlighted processes uniquely activated by TNFα or IL-1β [20]. The effects of IL-1β treatment on Cx43 have yielded controversial results in the literature. For instance, an upregulation has been described in urothelial cells [21], while a downregulation has been observed in astrocytes and cardiomyocytes [22,23]. In Kono et al., IL-1β induces an increase in Cx43 protein expression both at the gene and protein level in urothelial cells. No clear explanation is provided for the mechanism underlying the CX43 increase by IL-1β treatment. However, this increase was shown to be reduced by the anti-inflammatory flavonoid Nobiletin, whose action has been reported to involve the inhibition of the NF-κB pathway and the suppression of IL-6 and TNFα signaling [21]. Differently, in cardiomyocytes and astrocytes, the decrease in Cx43 expression and function was ascribed to the activation of p38 and the MAPK signaling pathway [22,23]. The unclear impact of IL-1β alone on modulating Cx43 expression prompts us to focus our attention on the effects of TNFα and its underlying mechanisms. The confirmation of TNFα efficacy in activating our primary cultures is evidenced by the upregulation of *MMP-3* and *MMP-13* gene expression [Figure 2B,C, Supplementary Figure S4]. The induction of MMPs, crucial for ECM disruption and type II collagen cleavage, by TNFα in CH has been previously demonstrated in our laboratory [19] and is well-documented in the literature for OB as well [20]. MMPs can be involved in Cx43 degradation and cleavage [24]. However, in our previous investigation, we did not detect the above-mentioned modifications in TNFα-treated CH [11]. CH and OB display disparate reactions to cytokine double treatment, with Cx43 down-modulation evident solely in chondrocytes. The differing outcomes may stem from various factors, including the distinct mechanisms underlying TNFα-mediated Cx43 reduction in the two cell types. Unlike CH, which did not exhibit a modification in *GJA1* expression following TNFα stimulation, OB showcased reduced Cx43 gene expression. The transcriptional modification of Cx43 by TNFα was previously documented by Tacheau [15] in keratinocytes, which also observed a reduction in its protein level. In addition, we checked the possible proteasome degradation of Cx43. Specifically, the co-administration of TNFα and a proteasome inhibitor partially prevented the reduction in Cx43 expression in OB. This suggests that in this cell type, TNFα acts at both the transcriptional level and, to a lesser extent, the proteasome level. The reduction in GJA1 expression could be associated with the activation of signaling pathways. For example, in astrocytes, Cx43 expression was downregulated by pathways involving NF-κB and PI3 kinase that are known to be both activated by TNFα [25]. Moreover, GJA1 expression might also by decreased via activation of JNK signaling, as identified in HaCat keratinocytes [15]. The connection between TNFα and proteasome activation, leading to the degradation of Cx43, has been noted in human corneal fibroblasts by Kimura et al. [16] and confirmed in chondrocytes, too [11]. TNFα has been shown to activate the proteasome in different cell types. In skeletal muscle cells, TNFα can promote activities and the contents of the 26S proteasome and the 19S regulator [26]. In nonprofessional antigen-presenting cells (APCs), TNFα increased the expression of catalytic immunoproteasome subunits, the immunomodulatory proteasome activator PA28α, the TAP1/TAP2 heterodimer and the total pool of MHC class I heavy chains [27]. In our previous study on CH, TNFα increased the levels of two components of the 20 Score proteasome complex (PSMA6 and PSMB4), which is a component of the proteasome regulatory system (PSMD10) and of the ubiquitin-conjugating enzyme E2 K (UBE2K) [11]. Additionally, there is evidence supporting the role of proteasome activity in the progression of osteoarthritis [28,29,30]. For these reasons, proteasome may play a critical role in mediating the observed effects. IL-1β effects on the ubiquitin-proteasome system are less documented. Whether the different effects mediated by the two cytokines rely on the different degrees of proteasome activation could be an interesting topic for future investigations. This provides valuable insights into the complex regulatory mechanisms involved in cytokine-induced alterations in Cx43 expression in OA-related cell types. The minor presence or even the absence of Cx43 has been linked to the delayed mineralization of the skeleton and decreased osteoblastic differentiation in vitro [31,32]. Li et al. [33] underscored the essential role of gap junctions in maintaining bone homeostasis and survival, as demonstrated by increased osteocyte apoptosis in the cortical bone of osteocyte-specific Cx43-deficient mice, leading to impaired bone strength. Cx43 also influences bone physiology through mechanisms beyond its channel activity, exerting regulatory control over transcription [34]. Previous evidence demonstrates that Cx43, particularly its carboxy tail, can translocate into the nucleus and acts as a transcriptional regulator, influencing cell growth and differentiation [7,35]. We detected Cx43 signal in osteoblasts nuclei, suggesting the potential for investigating its nuclear functions within this specific cell type. To explore the relevance of these findings in a more physio-pathological context, we use the synovial fluid from an OA patient to treat CH monolayers and cartilage explants. The decision to utilize synovial fluid (SF) instead of focusing solely on inflammatory cytokines like TNFα and IL-1β stems from the recognition that OA onset involves a complex interplay of various molecules. 

Normally, cartilage is regularly exposed to synovial fluid, which serves as both a biological lubricant and a biochemical reservoir facilitating the passage of nutrients and regulatory cytokines [36]. However, in conditions like osteoarthritis, the synovial fluid also contains a diverse range of compounds implicated in inflammation and catabolism, such as IL6, IFNγ, MMPs, TNFα and IL-1β. These compounds, produced by macrophages in response to tissue particles, debridement and microcrystals, start the inflammatory cascade, consequently impacting chondrocyte behavior [37]. Recent studies by Ragni et al. [38] have comprehensively characterized SF from OA patients and subsequent research [39] has investigated its impact on the gene expression profile of chondrocytes. Relevant to Cx43, our results show that the treatment of CH with SF significantly reduces both gene and protein expression. This suggests a distinct mechanism of Cx43 modulation between SF and TNFα, where the effect of TNFα is associated with proteasome degradation rather than modulation of gene expression. 

The final stage of our research involves evaluating the response of CH considering the entire tissue context, as represented by cartilage explants (CE). Chondrocytes reside within lacunae surrounded by a matrix composed primarily of collagen fibers and proteoglycans, which constitute most of the cartilage structure. Our findings indicate a reduction in Cx43 expression in the cartilage explants from the TNFα treatment. This is particularly relevant, especially considering the difference between 2D culture conditions, with high cell density and great cell-to-cell communication and the natural environment in which CHs resides (low cell density, reduced cell-to-cell communication). When exposing CE to synovial fluid, a similar decrease in Cx43 expression is observed. In the literature, the effect of synovial fluid on chondrocytes cultures and the cartilage explant is variable, and is also dependent on the pathology grade of the SF donor [39,40,41,42]. In our experimental setup, we observed no expression of COX-2 during culture with SF, in contrast to TNFα (Supplementary Figure S5). This suggests that SF may not elicit the same response as inflammatory cytokines. Further investigations are warranted to elucidate the effectors within synovial fluid that reduce Cx43 expression in both 2D chondrocytes and cartilage explants, together with clarifying the role of the modulation of GJA1 expression and/or proteasome involvement in cartilage explants.

The role of Cx43 in OA remains under investigation, with conflicting published data. While some studies suggest Cx43 overexpression in the OA cartilage [43,44], others highlight its protective role in maintaining structural integrity and function [10]. To address this question, it would be interesting to investigate Cx43 modulation using an OA animal model that closely mimics human conditions. This would involve using a large animal model (to replicate the load on articular tissues) and employing more appropriate techniques, such as immunohistochemistry (IHC) and mass spectrometry. Another interesting aspect to investigate might be the quantification of Cx43 in different joint tissues (such as cartilage, subchondral bone and synovial membrane) and its correlation with age, BMI, diagnosis and pathology grade. Moreover, the effect of different inflammatory stimuli on other cell types and tissues involved in joint pathophysiology, such as the synovial membrane and synoviocytes, would also be an interesting topic. In this context, our preliminary results on synovial cells confirm the effect of TNFα on Cx43 reduction in cells isolated from the synovial membrane (Supplementary Figure S6). Considering that different cell types reside in the synovial membrane, further steps should investigate in which cell types the reduction occurs. Finally, a longer exposure to inflammatory stimuli should be considered in order to better mimic chronic diseases such as osteoarthritis. This could be accomplished by a longer culture of ex vivo explants or 3D systems, with or without the use of scaffolds. To achieve this, the optimization of culture conditions (possibly including the use of bioreactors) should be performed to ensure cell viability throughout the prolonged culture period. A longer exposure in 2D culture could also be utilized; however, the dedifferentiation of cells could be a drawback of this approach. Our study contributes novel insights into the complex modulation of Cx43 in musculoskeletal diseases, especially regarding inflammatory joint disorders.

## 4. Materials and Methods

### 4.1. Cell and Cartilage Explant Isolation and Cultures

Articular chondrocytes (CH), cartilage explants (CE) and osteoblasts (OB) were isolated from the femoral heads of patients diagnosed with hip arthritis unresponsive to conservative therapy and classified as grade III osteoarthritis according to the Kellgren–Lawrence classification system [45] who underwent total hip replacement surgery at IRCCS Galeazzi–Sant’Ambrogio Hospital. All samples were obtained with patient-informed consent and ethical approval (TENET–IRCCS Ospedale San Raffaele ethics committee, approval number 38/int/2022). The donor features are indicated in Table 1.

For CH isolation and cartilage explants, cartilage specimens were excised with a scalpel from macroscopically intact regions of the femoral head, avoiding arthritic lesions. The selected portions had a polished surface, translucent appearance and semi-white cartilage, while areas displaying calcified cartilage were deliberately excluded. 

For CH isolation, cartilage was shredded with the scalpel and digested overnight with 1.5 mg/mL collagenase II (Worthington Biochemical Corporation, Lakewood, NJ, USA). The solution was filtered with a 100 µm cell strainer and CH were plated at a density of 10^4^ cells/cm^2^. CEs were obtained from cartilage using a surgical punch (4 mm) and cultured in a 12-well plate (10 explants/well). The cartilage explants model was characterized and described in our recent work [46]. Moreover, the histological evaluation of cartilage explants has been added as Supplementary Figure S7. CH and CE were maintained in a medium consisting of high-glucose DMEM supplemented with 10% FBS (HyClone, Euroclone, Pero, Italy, 2 mM glutamine, 50 μg/mL streptomycin, 50 U/mL penicillin and 110 μg/mL sodium pyruvate. For osteoblasts isolation, the trabecular bone was excised from the mid-deep area of the femoral head, then minced into fragments with a scalpel, washed with PBS (Phosphate-Buffered Saline, Euroclone, Pero, Italy) and vortexed several times in order to remove residual adipose and/or hematopoietic tissue and to promote the removal of debris and contaminant tissues. Bone chips were then placed in 60 mm Petri dishes and cultured in a complete medium (cDMEM) composed of high-glucose DMEM supplemented with 10% FBS (Euroclone, Pero, Italy), 2 mM L-glutamine, 50 U/mL penicillin, 50 μg/mL streptomycin 100 nM dexamethasone, 50 µg/mL ascorbic acid and 10 mM β glycerophosphate. Cells were maintained at 37 °C in a humidified atmosphere containing 5% CO_2_. 

### 4.2. Collection of Synovial Fluid

The synovial fluids (SF) of patients with osteoarthritis were collected and centrifuged at 16,000× *g* for 10 min at 4 °C [39]; the supernatants were stored at −80 °C until use. Treatments were performed with SF pools obtained from 13 to 15 patients, in order to minimize the inter-donor variability, as previously performed [39,47]. Donor features are summarized in Table 2.

### 4.3. Treatments

At first passage, upon reaching confluence, OB and CH were treated with TNFα (10 ng/mL) and/or IL-1β (1 ng/mL) for 1 and 3 days. One week after isolation, CE were treated with TNFα (10 ng/mL) for 3 days. Treatments were performed in cDMEM with 1% FBS. CH and CE were exposed at SF diluted 1:2 in cDMEM 1% FBS for 3 days. For specific experimental settings, the inhibitor of the proteasome MG132 at 1 µM was administered for 1 h prior to TNFα treatment.

### 4.4. Western Blotting

CH and OB were lysed in 50 mM Tris-HCl (pH 7.5), 150 mM NaCl, 1% NP-40 and 0.1% SDS with added protease inhibitor cocktail (PIC) and 2 mM PMSF. After an incubation in ice for 30 min and three freeze–thaw cycles, lysates were centrifuged at 14,000× *g* for 10 min at 4 °C. The protein extraction of CE was performed following TRIzol protocol. The proteins were resuspended in 100 µL of 1% SDS with inhibitors of proteases. Following centrifugation at 14,000× *g* for 10 min at 4 °C, supernatants were stored at −20 °C until use. The protein content was quantified through BCA assay (Thermo Fisher Scientific, Waltham, MA, USA) and 5–10 µg of proteins for each sample were analyzed by 10% SDS-PAGE and Western Blotting using standard protocols. Primary antibodies raised against Connexin 43 C-terminal (rabbit anti-Connexin43; #3512, Cell Signaling, Danvers, MA, USA, dilution ratio 1:1000) and β-actin (mouse anti-β-actin A2228, Merck, Darmstadt, Germany, dilution ratio 1:5000) were incubated at 4° overnight and at room temperature for 1 h, respectively. Specific signals were revealed after the incubation with the appropriate secondary antibodies conjugated to horseradish peroxidase followed by detection with ECL Westar Supernova (Cyanagen, Bologna, Italy). Signals were acquired using a Chemidoc Imaging System (Bio-Rad, Milan, Italy) and densitometry was quantified through Image Lab Software version 6.1 (Bio-Rad, Milan, Italy). To normalize target protein expression, the band intensity of each sample was divided by the intensity of the loading control protein ACTB. Then, the fold change was calculated by dividing the normalized expression from each lane by the normalized expression of the control sample (CTR = 1).

### 4.5. Gene Expression Analysis

One day after the treatments, CH and OB were lysed and RNA was extracted using an RNeasy Micro kit (Qiagen, Hilden, Germany). DNase I treatment (15 min) was performed to eliminate genomic contamination. RNA from CE was extracted with TRIzol following standard procedures and the genomic DNA was removed using DNase I, Amplification Grade (cat. N. 18068015, Thermo Fisher Scientific, Waltham, MA, USA). cDNA was synthetized with the High-Capacity Reverse-Transcription Kit according to the manufacturer’s protocol (Thermo Fisher Scientific, Waltham, MA, USA). The expression of *GJA1*, *MMP3*, *MMP13* and *TBP* were quantified via RT-qPCR using TaqMan technology (*GJA1:* Hs00748445_s1; *MMP3*: Hs00968305_m1; *MMP13*: Hs00233992_m1, *TBP*: Hs00427620_m1) at the QuantStudio Real-Time PCR systems (Thermo Fisher Scientific, Waltham, MA, USA). The data were analyzed with the 2^−∆∆Ct^ method [48].

### 4.6. Immunofluorescence

OB were seeded on 1 cm diameter glass coverslips at a density of 10^4^ cells/cm^2^. The day after, cells were fixed in 4% paraformaldehyde, permeabilized with 0.1% Triton X-100 and a double staining for Cx43 (primary antibody #3512, Cell Signaling, Danvers, MA, USA, dilution ratio 1:100; secondary antibody conjugated to AlexaFluor 488, Ab150073 Abcam, Cambridge, UK, dilution ratio 1:1000) and β tubulin (primary antibody T7815, dilution ratio 1:500; secondary antibody conjugated to AlexaFluor 488, Ab175473 Abcam, Cambridge, UK, dilution ratio 1:1000) was performed following standard procedures. OB were analyzed using the confocal laser scanning microscope TCS SP8 (Leica Microsystems CMS GmbH, Wetzlar, Germany). Images were acquired with a 63× objective and analyzed using Fiji software (ImageJ 1.51).

### 4.7. Statistics

Statistical analysis was performed using a paired t-test or one-way analysis of variance (ANOVA) using Tukey’s post hoc test. All the analyses were performed using Prism 9 (GraphPad Software, La Jolla, CA, USA). Data are expressed as mean ± SD and differences were considered significant at *p* ≤ 0.05.

## Figures and Tables

**Table 1:** Summary of donor characteristics for chondrocytes, osteoblasts and cartilage explants. BMI, body mass index.

	Age		Sex		BMI
	Range	Mean ± SD	Female	Male	Mean ± SD
Cartilage explants (*n* = 8)	60–88	72 ± 10	2	6	27.5 ± 4.3
Osteoblasts (*n* = 11)	53–83	70 ± 10	3	8	26.2 ± 3.2
Chondrocytes (*n* = 11)	53–83	66 ± 10	5	6	27.5 ± 4.3

**Table 2:** Summary of donor characteristics for synovial fluids. BMI, body mass index.

	Age		Sex		BMI
	Range	Mean ± SD	Female	Male	Mean ± SD
Pool 1 (*n* = 14)	50–88	69 ± 11	7	7	26.0 ± 3.9
Pool 2 (*n* = 13)	50–88	70 ± 10	6	7	26.7 ± 4.6
Pool 3 (*n* = 15)	42–88	68 ± 14	7	8	26.5 ± 4.5

## Data Availability

Data used to support the findings of this study will be uploaded in Zenodo repository upon publication.

## Supplementary Materials

The supporting information can be downloaded at: https://www.mdpi.com/article/10.3390/ijms25158547/s1.
