# Supplementary material for: Connexin 43 Modulation in Human Chondrocytes, Osteoblasts and Cartilage Explants: Implications for Inflammatory Joint Disorders
Source: Int J Mol Sci. 2024 Aug 5;25(15):8547. doi: 10.3390/ijms25158547 (PMC11313680; doi:10.3390/ijms25158547)
Supplement: Supplementary file 1 [file ijms-25-08547-s001.zip › ijms-3093642-supplementary.pdf]

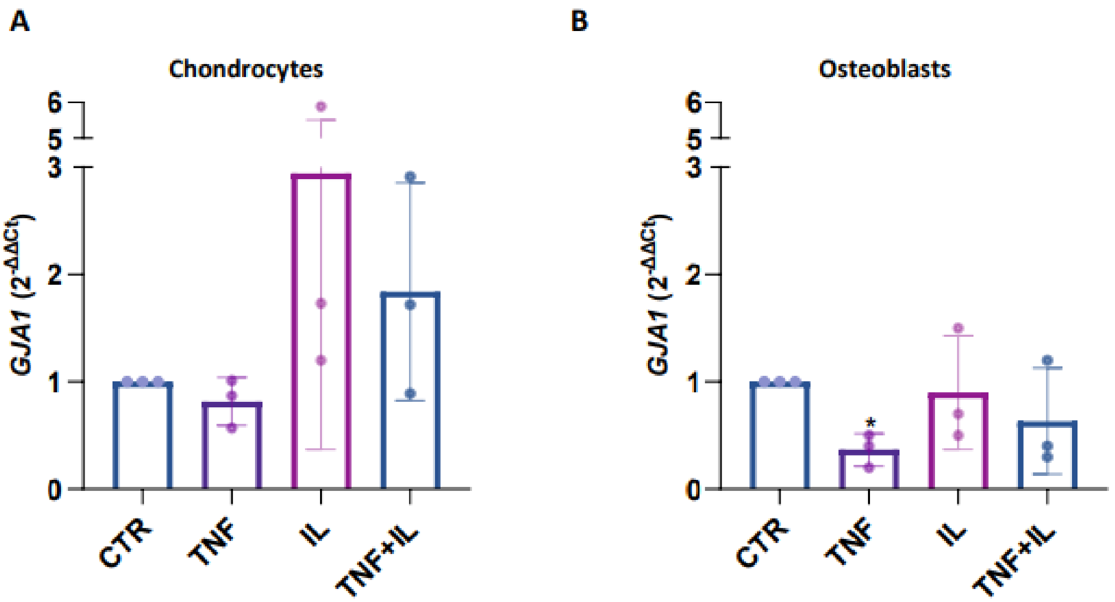

Supplementary Figure S1. A-B. Gene expression of GJA1 in TNFα and/or IL-1β-stimulated CH (**A**) and OB (**B**) at day 1 analyzed by real-time PCR. Data (n=3 independent experiments/donors) are expressed as $2^{-\Delta\Delta Ct}$ (TBP was used as a housekeeping gene). Data are shown as mean ± SD. Statistical analysis was performed by one-way analysis of variance (ANOVA) using Tukey's post hoc test. Significance vs CTR is shown as *$p<0.05$.
